# On the use of QDE-SVM for gene feature selection and cell type classification from scRNA-seq data

**DOI:** 10.1371/journal.pone.0292961

**Published:** 2023-10-19

**Authors:** Grace Yee Lin Ng, Shing Chiang Tan, Chia Sui Ong

**Affiliations:** Faculty of Information Science and Technology, Multimedia University, Bukit Beruang, Melaka, Malaysia; State University of New York at Oswego, UNITED STATES

## Abstract

Cell type identification is one of the fundamental tasks in single-cell RNA sequencing (scRNA-seq) studies. It is a key step to facilitate downstream interpretations such as differential expression, trajectory inference, etc. scRNA-seq data contains technical variations that could affect the interpretation of the cell types. Therefore, gene selection, also known as feature selection in data science, plays an important role in selecting informative genes for scRNA-seq cell type identification. Generally speaking, feature selection methods are categorized into filter-, wrapper-, and embedded-based approaches. From the existing literature, methods from filter- and embedded-based approaches are widely applied in scRNA-seq gene selection tasks. The wrapper-based method that gives promising results in other fields has yet been extensively utilized for selecting gene features from scRNA-seq data; in addition, most of the existing wrapper methods used in this field are clustering instead of classification-based. With a large number of annotated data available today, this study applied a classification-based approach as an alternative to the clustering-based wrapper method. In our work, a quantum-inspired differential evolution (QDE) wrapped with a classification method was introduced to select a subset of genes from twelve well-known scRNA-seq transcriptomic datasets to identify cell types. In particular, the QDE was combined with different machine-learning (ML) classifiers namely logistic regression, decision tree, support vector machine (SVM) with linear and radial basis function kernels, as well as extreme learning machine. The linear SVM wrapped with QDE, namely QDE-SVM, was chosen by referring to the feature selection results from the experiment. QDE-SVM showed a superior cell type classification performance among QDE wrapping with other ML classifiers as well as the recent wrapper methods (i.e., FSCAM, SSD-LAHC, MA-HS, and BSF). QDE-SVM achieved an average accuracy of 0.9559, while the other wrapper methods achieved average accuracies in the range of 0.8292 to 0.8872.

## Introduction

Single-cell RNA sequencing (scRNA-seq) generates the expression profile of transcripts for every single cell in a given population [1], and provides high-resolution insights for current biomedical studies. Unlike bulk RNA sequencing (RNA-seq) that provides the average expression of all cells, scRNA-seq treats cells individually to study the differences in each cell [2]. Questions as to which cells can be effectively targeted in studies such as cancer treatments or drug designs could be answered by analyzing the transcriptomic data from scRNA-seq [3, 4]. These analyses include cell type or cell state identification [5, 6], cell clustering [7], differential expression [8], spatial transcriptomics [9], and others [10, 11]. Identification of cell type, including cell state and cell cycle stage, is one of the fundamental tasks in scRNA-seq analyses. It is a key step in making sense of the data to facilitate downstream interpretations such as differential expression and trajectory inference [12, 13]. There are mainly two ways to identify cell types: classification and clustering [6]. Clustering is useful to identify novel and rare cell types. As scRNA-seq studies progress, many data are accumulated and tagged with cell types by referring to expert knowledge, and this leads to the introduction of cell type classification studies [14].

The advancements in single-cell sequencing technologies and protocols now enable millions of cells to be sequenced [15]. However, downstream analysis interpretations would not be accurate if technical variabilities, such as batch effects and biological factors are left unaccounted for [16]. Therefore, gene selection plays a crucial role in selecting a smaller but relevant set of genes for carrying out informative scRNA-seq analyses. Gene selection, also known as feature selection in data science, is a typical task for identifying salient features from a high-dimensional scRNA-seq dataset that comprises noises. Generally speaking, feature selection methods are categorized into filter-, wrapper-, and embedded-based approaches [17]. The filter-based feature selection methods are generally fast as they rank and filter the features directly based on a metric quantifying data characteristics such as information, distance, or correlation [18–20]. The wrapper-based feature selection methods involve wrapping a feature subset selection algorithm (that searches for a good subset of features) around a learning algorithm (that evaluates the goodness of feature subsets) to find optimal and relevant features [21]. Embedded-based methods, on the other hand, integrate feature selection in the process of learning while avoiding long computational time [17, 18].

A number of gene selection algorithms and tools have been introduced in the literature for scRNA-seq cell type identification. Among the aforementioned three feature selection approaches, the filter-based approach is the most widely used [22] in finding a subset of useful gene features from scRNA-seq datasets. An example of the tool from this approach is scmap [23], which relies on dropouts, highly variable genes, and random selection to select genes for projecting cell types across datasets. Seurat [9] is another impactful filter-based method that selects highly variable genes to decode the spatial heterogeneity of the scRNA-seq gene expression data. CaSTLe [24] is a filter-based method that selects genes for cell type, cell state, or cell cycle labeling according to the mean expression of features, mutual information between features and class, and inter-feature correlation. scClassify [25] selects genes that discriminate among cell types based on differentially expressed genes, differential variable genes, differentially distributed genes, differentially proportioned genes, and bimodally distributed genes. COMET [26] is another tool designed for selecting marker genes that differentiate cells. It uses a hypergeometric test to evaluate the gene enrichment in a particular cell cluster. Other filter-based methods include concepts such as entropy [27], analysis of variance [28], or co-expression [29] for cell-type-specific gene selection.

Besides filter-based methods, a number of embedded-based gene selection methods are also available for scRNA-seq cell type identification. For example, RFCell [30] uses data permutation to generate negative samples, followed by a random forest to evaluate the importance of genes in cell type identification and biological interpretation. NS-forest [31] uses a random forest to select gene features. The features are further filtered by referring to a binary expression score. Sen Puliparambil et al. [32] introduced a method to select a set of discriminative genes using multiple penalization models. Other embedded-based gene selection methods incorporate models such as logistics regression (LR) [33], autoencoder [34], or deep learning [35].

In the literature, the number of tools that are developed in the wrapper approach to perform scRNA-seq gene selection is relatively few. Information gain ratio and genetic algorithm with dynamic crossover (IGRDCGA) [36] is one of the wrapper-based methods used for scRNA-seq gene selection. Information gain ratio is used as a measurement to eliminate irrelevant genes. Genetic algorithm of dynamic crossover wrapped with k-means clustering is then utilized to select genes and improve cell classification. Another wrapper-based tool is single-cell feature selection method based on convex analysis of mixtures (FSCAM) [37], which uses convex analysis of mixture (CAM) and fruit fly optimization algorithm (FFOA) as a wrapper to search for genes enriched in specific cell types. FSCAM applies prefiltering of genes based on zero read counts, mean expression, and dropouts. The differentially expressed genes selected by FSCAM for cell type clustering have superior clustering performance among methods from the other categories. On the other hand, Feature Selection via Genetic Algorithm (FSGA) [38] is one such method that uses classification learning algorithm for evaluating the goodness of the selected features. *k*-nearest neighbours (KNN) classifier wrapped by a genetic algorithm is used in this work. FSGA also emphasizes the biological relevancy of the selected features.

Despite superior performances of these methods, to the best of our knowledge, there is a lack of benchmarking studies utilizing wrapper-based gene selection methods in the literature. The wrapper-based methods have shown promising results in other fields [39–41]; however, the effectiveness of this feature selection category in selecting gene features from scRNA-seq datasets has yet been extensively investigated. In fact, both learning algorithm and the feature subset searching algorithm are necessary in selecting optimal gene sets that are useful to describe cell types. Another note is that, many existing wrappers resort to clustering as the learning component in the wrapper-based feature selection process [36, 37]. With efforts from the pioneers, Cell Ontology [42] is established to provide an updated list of cell types. This comprehensive ontology is referred to create a large number of labeled scRNA-seq datasets. The main interest of our work is to investigate the effectiveness of a classification-based wrapper method in selecting gene subsets from the labeled scRNA-seq datasets to classify cell types. In this regard, a quantum-inspired differential evolution (QDE) wrapped with a classification algorithm is utilized to select genes from the annotated scRNA-seq datasets.

The rest of the content in this paper is divided into several sections. The “Materials and Methods” section explains the datasets, gene selection methods, and experimental workflow applied in this study. The “Results” section compares the performance of different feature selection methods in the experimental study, while the “Discussion” section presents the analyses and findings from the study. Finally, the “Conclusion” section summarizes the work.

## Materials and methods

### Datasets

Twelve popular scRNA-seq datasets from the public portals are used in this study. These datasets comprise the records of single cells from various cell types or cell cycle stages. The twelve datasets selected for this study contain single cells from human and mouse samples. They can be divided into three broad categories, namely developmental, metabolic diseases, and connective tissues. Four datasets from human [43] and mouse [44–46] embryos at different development stages belong to the developmental category. Five datasets from healthy or type 2 diabetic pancreas tissues belong to the metabolic diseases category wherein four of them are from humans [47–50], and one from mice [50]. Another three datasets from the brain cells (one from humans [51] and two from mice [52, 53]) belong to the connective tissues category. Each dataset is named based on the isolated tissue, organism, author’s first name, and year of dataset publication, separated by underscores. A summary of the dataset information is provided in Table 1.

**Table 1:** Summary of scRNA-seq datasets used in this study.

No.	Dataset Name	No. of cells (samples)	No. of genes (features)	No. of cell types (classes)	Normalization ^a^	Ref.
1	Embryo_Human_Yan_2013	124	14514	8	-	[43]
2	Embryo_Mouse_Biase_2014	56	25737	5	FPKM	[44]
3	Embryo_Mouse_Goolam_2016	124	41480	5	-	[46]
4	Embryo_Mouse_Deng_2014	304	22958	12	-	[45]
5	Pancreas_Human_Lawlor_2017	617	26616	7	TPM	[47]
6	Pancreas_Human_Xin_2016	1600	39852	4	RPKM	[49]
7	Pancreas_Human_Segerstolpe_2016	2127	26272	11	-	[48]
8	Pancreas_Human_Baron_2016	8569	20125	14	-	[50]
9	Pancreas_Mouse_Baron_2016	1886	14878	13	-	[50]
10	Brain_Human_Darmanis_2015	466	22085	9	-	[51]
11	Brain_Mouse_Zeisel_2015	3005	19972	7	-	[52]
12	Brain_Mouse_Tasic_2016	1727	24150	49	-	[53]

^a^ FPKM: fragments per kilobase per million mapped fragments, TPM: transcript per million, RPKM: reads per kilobase per million mapped reads.

In this study, a minimal preprocessing is applied to the data by excluding cells with ambiguous labels such as “None/Other” in Pancreas_Human_Lawlor_2017; “not applicable”, “unclassified endocrine cell”, “unclassified cell”, and “co-expression cell” in Pancreas_Human_Segerstolpe_2016. The absolute or normalized gene counts are retained as in the original dataset sources. No other normalization techniques have been applied to the data with a purpose to evaluate the performance of the gene selection method in selecting gene features from the datasets.

### Classification-based QDE

In this study, a wrapper-based feature selection by a metaheuristic approach is employed, where QDE is utilized as a feature subset searching algorithm to select the genes that characterize cell types. A classification algorithm is used to quantize the goodness of these gene subsets in classifying cell types. QDE is a metaheuristic algorithm based on differential evolution (DE) that uses quantum computing in the feature subset initialization process [54]. It has been applied in various domains from numerical optimization, to discrete optimization such as feature selection for biomedical and radar signaling classifications [55], as well as biomarker selection [56]. In these discrete optimizations, QDE selects features from low-dimensional datasets containing few features. A DE method is considered in this work because it is an accurate metaheuristic method [57]. On the other hand, quantum-based metaheuristic variants, including QDE, could search for optimal solutions at a high convergence rate [54, 58, 59]. In view of these advantages, QDE is applied in this study to search for optimal feature subsets from the high-dimensional scRNA-seq datasets.

The QDE feature selection process begins by initializing 30 feature subsets (candidate solutions), which is the same setting as mentioned in Srikrishna et al. [55]. Assume that a dataset consists of *D* gene features; each candidate solution is made up of *D* binary bits, where a state of 1 indicates that the corresponding feature is selected as a candidate feature, and a state of 0 indicates that it is not included in the solution. The state of each feature is determined by observing a quantum bit. This quantum-based initialization process is adopted from the work by Srikrishna et al. [55]. The first 30 candidate solutions are generated through a serial process of quantum-based initialization and observation to form an initial population.

The fitness (goodness) of these 30 candidate solutions in representing cell types will be evaluated using a classifier. In this research, five machine learning (ML) classifiers are employed to determine the most suitable classification model for handling scRNA-seq data. Four of them are coded using the scikit-learn library [60], namely LR, decision tree (DT), support vector machine (SVM) with linear kernel, and SVM with radial basis function (RBF) kernel. The fifth classifier is the extreme learning machine (ELM), a fast neural network with a single hidden layer [61] that is available on GitHub [62]. The default hyperparameter settings of five classifiers are listed in Table 2. Notably, the hyperparameter settings of five classifiers are not fine-tuned; they are relaxed at the default settings. The purpose is to make a fair performance comparison of QDE with these ML classifiers using twelve datasets. As most of the datasets are imbalanced, F1-score, which is a harmonic mean of precision and recall with a range between 0 to 1 [63], is used as one of the computing elements of the fitness score. This is to evaluate the goodness of a gene features subset in classifying cell types. The fitness score for the classification-based QDE is based on two elements, (1) classification performance, i.e., the F1-score, and (2) the number of selected genes. This is to ensure the feature subsets discovered are able to classify cells accurately using a smaller number of gene features. The fitness score of a candidate feature set is defined as follows:

$$Fitness\;score=F1+\left(1-\frac{N}{D}\right)$$ (1)

where *F1* is the F1-score of the feature set, *N* is the number of selected genes, and *D* is the total number of genes before selection.

**Table 2:** Hyperparameter settings applied to each ML classifiers.

ML Classifier	Hyperparameter settings	Source	Ref.
LR	penalty = "l2", tol = 1e-4, C = 1.0, fit_intercept = True, intercept_scaling = 1, class_weight = None, random_state = None, solver = "lbfgs", max_iter = 100, multiclass = "auto", warm_start = False, n_jobs = None, l1_ratio = None	sklearn.linear_model.LogisticRegression()	[60]

DT	criterion = "gini", splitter = "best", max_depth = None, min_samples_split = 2, min_samples_leaf = 1, min_weight_fraction_leaf = 0.0, max_features = None, random_state = None, max_leaf_nodes = None, min_impurity_decrease = 0.0, class_weight = None, ccp_alpha = 0.0	sklearn.tree.DecisionTreeClassifier()	
SVM with linear kernel	C = 1.0, kernel = "linear", degree = 3, gamma = "scale", coef0 = 0.0, shrinking = True, probability = False, tol = 1e-3, cache_size = 200, class_weight = None, max_iter = -1, decision_function_shape = "ovr", break_ties = False, random_state = None	sklearn.svm.SVC()	
SVM with RBF kernel	Same as SVM with linear kernel except kernel = "rbf"		
ELM	n_hidden = 20, alpha = 0.5, rbf_width = 1.0, activation_func = "tanh", activation_args = None, user_components = None, regressor = None, binarizer = LabelBinarizer(-1, 1), random_state = None	elm.ELMClassifier()	[62]

Mutation and crossover of the population are performed before forming a new population. This process creates child solutions in which their performance is then compared with their respective parent solution. The mutation and crossover are performed at a rate of 0.8 out of 1 to explore for better candidate solutions in the large feature subspace while still exploiting the current solutions [64]. After generating the child population, both parent and child solutions will be considered in a selection process to form a new population for the next generation. The elitist selection strategy [55] is applied in this study. If the fitness score of the parent solution is lower than the elitism threshold, the corresponding child solution is selected for the next generation. However, if the fitness score of the child solution is lower than the parent solution, the parent solution remains in the population for the next generation. The elitism threshold is defined as follows:

$$Elitism\;threshold={F1}_{i}+(1-N_{i})$$ (2)

where *F*1_*i*_ is obtained from the mean F1-score of the initial population with an addition of 0.1 to allow improvement in the classification performance. *N*_*i*_ is an adjustable variable for the ideal portion of features to be selected. It is set to 0.01 throughout this study, i.e., 1% of the total number of features, to determine the best-performing feature subset.

**Fig 1:**
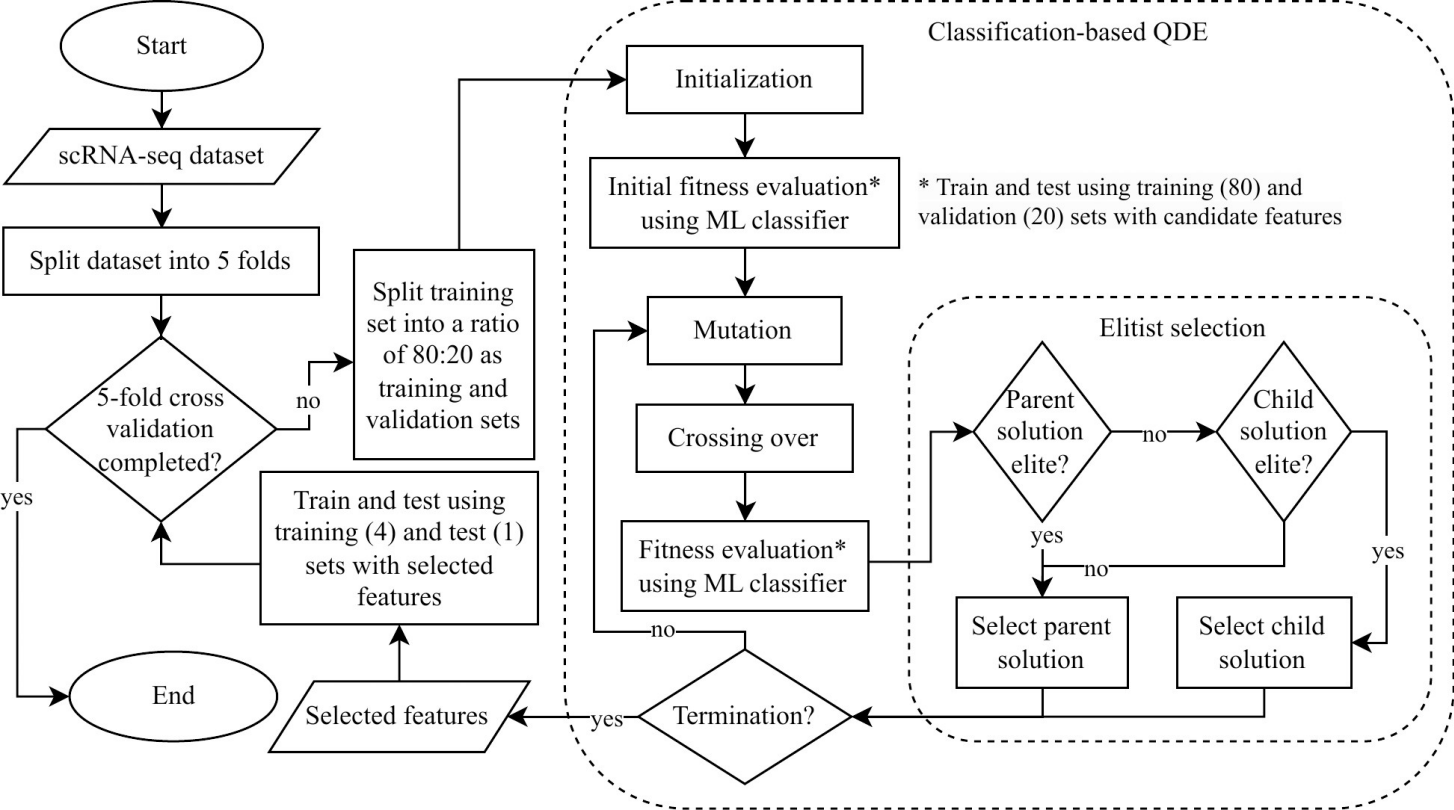
Flowchart of classification-based QDE and 5-fold cross-validation process. The fitness evaluation step is done based on the F1-score from a classification process. Five ML classifiers (LR, DT, SVM with linear and RBF kernel, as well as ELM) are tested for fitness evaluation in this QDE model. The performance of the feature subsets selected using classification-based QDE is evaluated in a 5-fold cross-validation process.

The process of feature subset evaluation, mutation, crossover, and selection is continued until reaching a specified number of generations. Since all datasets have a large number of features, the QDE is executed for 100 iterations so that the evolution process is carried out not too long to avoid overfitting results. The flowchart of the classification-based QDE is depicted in Fig 1.

### Experimental setup

The experiments are designed in two stages using a 5-fold cross-validation strategy: (1) in the first stage, the best classifier for wrapper QDE is determined; (2) in the second stage, the selected method from stage 1 is used to compare with the classification performance of the recent methods comprising different feature selection algorithms.

In the first stage, the experiment is conducted using QDE wrapped with five different classifiers using the hyperparameters settings listed in Table 2. Thus, five methods have been developed, namely QDE with LR (QDE-LR), QDE with DT (QDE-DT), QDE with SVM of linear kernel (QDE-SVM), QDE with SVM of RBF kernel (QDE-SVMrbf), and QDE with ELM (QDE-ELM). A 5-fold cross-validation is used in the experiment (Fig 1). Each dataset is divided into five portions. The function StratifiedKFold() from scikit-learn library [60] is applied in this work. A portion of the dataset is set as the test set. The remaining four portions are used in the feature selection process, which are further divided into training and validation sets using the function train_test_split() from scikit-learn library [60] with a ratio of 80:20. In each fold, the training set is used to train a group (or a population) of classifiers using different subsets of gene features (candidate solutions) provided by QDE, and the validation set is used to compute the fitness score of the candidate solution. The average accuracy, F1-score, number of selected features, and the total time taken for the five test sets on each method are recorded.

The second stage of the experiment is a comparison of classification performance between the best-performing QDE method chosen from the first stage and four recent methods with different feature selection algorithms [37, 65–67] that have also been developed by a wrapper approach. Unless specified, otherwise all hyperparameter settings of these recent methods are respectively referred from [37, 65–67].

One of the recent methods is FSCAM [37]. FSCAM applies a metaheuristic search strategy (i.e., FFOA) to find the optimal feature set. However, instead of a classification approach, FSCAM utilizes a clustering method (i.e., CAM) to evaluate the goodness of a feature subset. Another difference between the proposed classification-based QDE and FSCAM is that the latter imposes data preprocessing. FSCAM filters genes of zero read counts, dropouts, and genes of extremely high or low mean expression levels before conducting feature selection. In FSCAM, genes are modeled into a convex set. Identification of the vertices for the convex set corresponds to the identification of differentially expressed genes from the scRNA-seq data. This process is aided by optimization using FFOA. Genes that are exclusively expressed in a cell cluster (cell types) are preferred. FSCAM was originally an unsupervised method used to identify cell types. In this study, the genes selected from FSCAM are evaluated for their accuracy in cell type classification using a linear SVM. In our work, the hyperparameters of the SVM are in the same settings as the linear SVM from the best QDE method (i.e., QDE-SVM).

Apart from FSCAM, another three classification-based wrapper methods, namely hill-climbing-based social ski driver (SSD-LAHC) algorithm [65], mayfly-harmony search (MA-HS) [67], and binary sailfish (BSF) optimizer [66], are also included in the benchmark study. Notably, these three methods are general metaheuristic wrapper-based methods that have yet to be applied in the domain of scRNA-seq gene selection. In [65–67], the classifiers wrapping these algorithms are the KNN classifiers. The number of iterations and potential solutions per population of SSD-LAHC, MA-HS, and BSF are set to the same settings as those of the classification-based QDE (i.e., 100 iterations and 30 solutions per population) for aligning experimental setup in the benchmark study. All of the experiments have been conducted in a workstation with an Intel(R) Xeon(R) W-2195 CPU @ 2.30GHz and a RAM of 64GB.

## Results

### Stage 1: Comparison of classification-based QDE methods

Table 3 shows the classification performance of gene feature subsets selected by QDE with five different classifiers in terms of accuracy and F1-score. It can be seen that the QDE models with DT, SVM of RBF kernel, and ELM as the classifier perform with lower scores as compared to QDE with LR and linear SVM.

**Table 3:** Accuracy and F1-score from different QDE methods.

**(A) Accuracy**					
**Dataset**	**QDE-LR**	**QDE-DT**	**QDE-SVM**	**QDE-SVMrbf**	**QDE-ELM**
Embryo_Human_Yan_2013	0.9848	0.8860	0.9859	0.9138	0.7688
Embryo_Mouse_Biase_2014	0.9076	0.8567	0.9176	0.8226	0.5958
Embryo_Mouse_Goolam_2016	0.9682	0.8816	0.9668	0.7560	0.7212
Embryo_Mouse_Deng_2014	0.9263	0.7128	0.9249	0.7803	0.5800
Pancreas_Human_Lawlor_2017	0.9719	0.9650	0.9688	0.9326	0.8955
Pancreas_Human_Xin_2016	0.9972	0.9973	0.9974	0.9900	0.9844
Pancreas_Human_Segerstolpe_2016	0.9870	0.9641	0.9870	0.9341	0.9220
Pancreas_Human_Baron_2016	0.9774	0.9451	0.9776	0.9357	0.8498
Pancreas_Mouse_Baron_2016	0.9710	0.9274	0.9685	0.8749	0.8473
Brain_Human_Darmanis_2015	0.8905	0.7187	0.8721	0.8140	0.5871
Brain_Mouse_Zeisel_2015	0.9648	0.9044	0.9703	0.9541	0.7252
Brain_Mouse_Tasic_2016	0.7991	0.5694	0.8101	0.5211	0.2582
***Average accuracy***	***0*.*9455***	***0*.*8607***	***0*.*9456***	***0*.*8524***	***0*.*7280***
**(B) F1-score**					
**Dataset**	**QDE-LR**	**QDE-DT**	**QDE-SVM**	**QDE-SVMrbf**	**QDE-ELM**
Embryo_Human_Yan_2013	0.9838	0.8783	0.9843	0.9052	0.7503
Embryo_Mouse_Biase_2014	0.8931	0.8455	0.9062	0.7709	0.5640
Embryo_Mouse_Goolam_2016	0.9630	0.8749	0.9638	0.7051	0.6851
Embryo_Mouse_Deng_2014	0.9241	0.7052	0.9239	0.7694	0.5552
Pancreas_Human_Lawlor_2017	0.9716	0.9649	0.9681	0.9211	0.8760
Pancreas_Human_Xin_2016	0.9972	0.9973	0.9974	0.9894	0.9837
Pancreas_Human_Segerstolpe_2016	0.9864	0.9639	0.9863	0.9206	0.9110
Pancreas_Human_Baron_2016	0.9771	0.9448	0.9771	0.9302	0.8218
Pancreas_Mouse_Baron_2016	0.9701	0.9258	0.9679	0.8536	0.8224
Brain_Human_Darmanis_2015	0.8791	0.7155	0.8634	0.7881	0.5141
Brain_Mouse_Zeisel_2015	0.9647	0.9044	0.9703	0.9534	0.6947
Brain_Mouse_Tasic_2016	0.7945	0.5641	0.8058	0.4399	0.1819
***Average F1-score***	***0*.*9420***	***0*.*8570***	***0*.*9429***	***0*.*8289***	***0*.*6967***

When observing the time taken to complete the feature selection process (Table 4), QDE with DT and ELM are generally faster than the others (an average time of 10.41 hours and 7.55 hours respectively), QDE-SVM is slightly slower at an average time of 29.85 hours, followed by QDE-LR with an average time of 38.00 hours. QDE-SVMrbf is the slowest with an average time of 86.70 hours. As a fast and single-layered neural network, ELM has the shortest time of execution as compared to the other classifiers. However, the gene features subsets selected by QDE-ELM lead to the lowest accuracy (an average accuracy of 0.7280) and F1-score (an average F1-score of 0.6967) among all the other methods, especially for the non-pancreatic datasets. SVM with RBF kernel could help QDE to select genes with similar accuracy and F1-scores as DT, but the time required to complete the same number of iterations is the longest with QDE-SVMrbf. QDE-LR and QDE-SVM give a similar performance in terms of classification scores (around 0.94 accuracy and F1-score from both methods). Nevertheless, considering the time of execution, QDE-SVM is a better method as it gives high classification accuracy with a shorter training time duration (an average time of 29.85 hours) than QDE-LR (an average time of 38.00 hours). By referring to Table 5, the number of features can be reduced to around half of the original number for all methods as they are using the same QDE searching scheme.

**Table 4:** Time taken (in hours) for feature selection in different QDE methods.

Dataset	QDE-LR	QDE-DT	QDE-SVM	QDE-SVMrbf	QDE-ELM
Embryo_Human_Yan_2013	10.60	1.85	1.02	1.11	2.52
Embryo_Mouse_Biase_2014	9.45	2.04	3.20	2.91	2.76
Embryo_Mouse_Goolam_2016	28.48	3.47	4.50	4.68	4.94
Embryo_Mouse_Deng_2014	42.34	4.84	7.79	15.74	3.79
Pancreas_Human_Lawlor_2017	40.65	6.00	9.34	22.91	5.16
Pancreas_Human_Xin_2016	29.25	6.34	25.17	119.73	24.02
Pancreas_Human_Segerstolpe_2016	57.51	14.98	46.83	306.34	9.65
Pancreas_Human_Baron_2016	65.16	20.11	103.33	300.25	14.15
Pancreas_Mouse_Baron_2016	29.30	2.53	15.17	31.79	5.59
Brain_Human_Darmanis_2015	29.64	4.64	16.10	12.77	4.20
Brain_Mouse_Zeisel_2015	28.93	14.54	86.31	176.52	10.26
Brain_Mouse_Tasic_2016	84.72	43.58	39.45	45.61	3.59
***Average time (hours)***	***38*.*00***	***10*.*41***	***29*.*85***	***86*.*70***	***7*.*55***

**Table 5:** Number of selected features from different QDE methods.

Dataset	QDE-LR	QDE-DT	QDE-SVM	QDE-SVMrbf	QDE-ELM
Embryo_Human_Yan_2013	7152.09	7203.23	7148.93	7208.87	7243.65
Embryo_Mouse_Biase_2014	12744.13	12788.56	12733.49	12774.23	12857.65
Embryo_Mouse_Goolam_2016	20579.74	20683.09	20578.70	20674.29	20731.76
Embryo_Mouse_Deng_2014	11427.58	11476.44	11406.62	11434.92	11473.19
Pancreas_Human_Lawlor_2017	13247.88	13268.34	13251.16	13297.48	13297.39
Pancreas_Human_Xin_2016	19794.98	19809.37	19819.90	19846.75	19884.28
Pancreas_Human_Segerstolpe_2016	13054.79	13087.13	13055.55	13104.43	13120.73
Pancreas_Human_Baron_2016	9978.45	10008.35	9970.22	10041.50	10051.75
Pancreas_Mouse_Baron_2016	7380.60	7403.45	7385.53	7422.98	7426.81
Brain_Human_Darmanis_2015	11019.31	11023.87	11018.63	11023.45	11033.97
Brain_Mouse_Zeisel_2015	9920.98	9946.50	9916.18	9945.14	9967.94
Brain_Mouse_Tasic_2016	12058.17	12063.04	12059.45	12054.51	12078.05
***Average number of selected features***	***12363*.*23***	***12396*.*78***	***12362*.*03***	***12402*.*38***	***12430*.*60***

Before selecting the best QDE method, statistical test was performed. Since the results are not normally distributed, where they are largely skewed from the mean value, a non-parametric statistical test was applied. Friedman test [68] was chosen and performed for the results (accuracy, F1-score, number of features, and time taken) on the twelve datasets from five different QDE methods. The null hypothesis is that all of the methods have statistically similar results. A significance level of *α* = 0.05 was used. The *p*-values of all tests listed in Table 6 are lower than α, indicating that all null hypotheses are rejected, where at least one of the five methods is statistically different from the others in the aspects of classification performance, number of features, and time of execution.

**Table 6:** Friedman tests for 5 different QDE methods (QDE-LR, QDE-DT, QDE-SVM, QDE-SVMrbf, and QDE-ELM) in terms of accuracy, F1-score, number of features, and time taken.

Comparison	Test statistic	*p*-value	Hypothesis
Five QDE models (Accuracy)	42.29	1.45E-08	Rejected
Five QDE models (F1-score)	42.29	1.45E-08	Rejected
Five QDE models (Number of features)	36.33	2.47E-07	Rejected
Five QDE models (Time taken)	28.47	1.00E-05	Rejected

To identify how the methods are different from each other, post hoc tests were conducted using Holm’s procedure [69] for pairwise comparison. QDE-SVM is selected as the control algorithm as it showed the most advantages as discussed earlier. The post hoc tests also aimed to validate if QDE-SVM is a better method by showing statistically significant results from the other methods. The null hypothesis is that QDE-SVM is statistically similar to a compared method.

The results in Table 7 show that QDE-SVM achieves similar accuracy and F1-score with QDE-LR, where both of them are significantly more accurate as compared to the rest. The number of features selected by QDE-SVM and QDE-LR are also statistically equal. It can be inferred that QDE-LR and QDE-SVM are slightly better at selecting important features, as both of them have a relatively lower average feature number (Table 5). QDE-SVM requires a similar time duration as taken by QDE-DT, QDE-SVMrbf, and QDE-ELM. These test results show that QDE-SVM can achieve as good classification results as QDE-LR in a shorter time. Thus, QDE-SVM is selected as the best method in stage 1 for further comparison with other wrapper-based feature selection methods.

**Table 7:** Post hoc tests among different QDE methods (QDE-SVM as the control algorithm) using Holm’s procedure.

**(A) Accuracy of cell type classification using selected features**					
***i***	**QDE-SVM *vs*.**	***z***	***p*-value**	**Adjusted α**	**Hypothesis**
4	QDE-LR	-0.4743	0.6353	0.0500	Accepted
3	QDE-DT	-3.4785	0.0005	0.0250	Rejected
2	QDE-SVMrbf	-4.2691	0.0000	0.0167	Rejected
1	QDE-ELM	-6.7989	0.0000	0.0125	Rejected
**(B) F1-score of cell type classification using selected features**					
***i***	**QDE-SVM *vs*.**	***z***	***p*-value**	**Adjusted α**	**Hypothesis**
4	QDE-LR	-0.1581	0.8744	0.0500	Accepted
3	QDE-DT	-3.3204	0.0009	0.0250	Rejected
2	QDE-SVMrbf	-4.1110	0.0000	0.0167	Rejected
1	QDE-ELM	-6.6408	0.0000	0.0125	Rejected
**(C) Number of selected features**					
***i***	**QDE-SVM *vs*.**	***z***	***p*-value**	**Adjusted α**	**Hypothesis**
4	QDE-LR	0.1581	0.8744	0.0500	Accepted
2	QDE-DT	3.6366	0.0003	0.0167	Rejected
3	QDE-SVMrbf	3.4785	0.0005	0.0250	Rejected
1	QDE-ELM	6.1664	0.0000	0.0125	Rejected
**(D) Time taken for feature selection**					
***i***	**QDE-SVM *vs*.**	***z***	***p*-value**	**Adjusted α**	**Hypothesis**
1	QDE-LR	2.6879	0.0072	0.0125	Rejected
3	QDE-DT	-2.2136	0.0269	0.0250	Accepted
4	QDE-SVMrbf	1.8974	0.0578	0.0500	Accepted
2	QDE-ELM	-2.3717	0.0177	0.0167	Accepted

### Stage 2: Comparison of wrapper-based gene selection methods

The method introduced in this study is further compared with recent wrapper-based methods, including a clustering-based wrapper method (i.e., FSCAM), and three classification-based wrapper methods (i.e., SSD-LAHC [65], MA-HS [67], and BSF [66]). As mentioned in the “Materials and Methods: Experimental setup” section, the performances of features selected by FSCAM on test sets were determined using a linear SVM. This is to ensure the performance of FSCAM, originally an unsupervised cell type identification method, is comparable with other classification-based methods by using comparable metrics (i.e., accuracy and F1-score). In our work, the three classification-based wrappers were used with the original classifier (i.e., KNN) as in [65–67].

**Fig 2:**
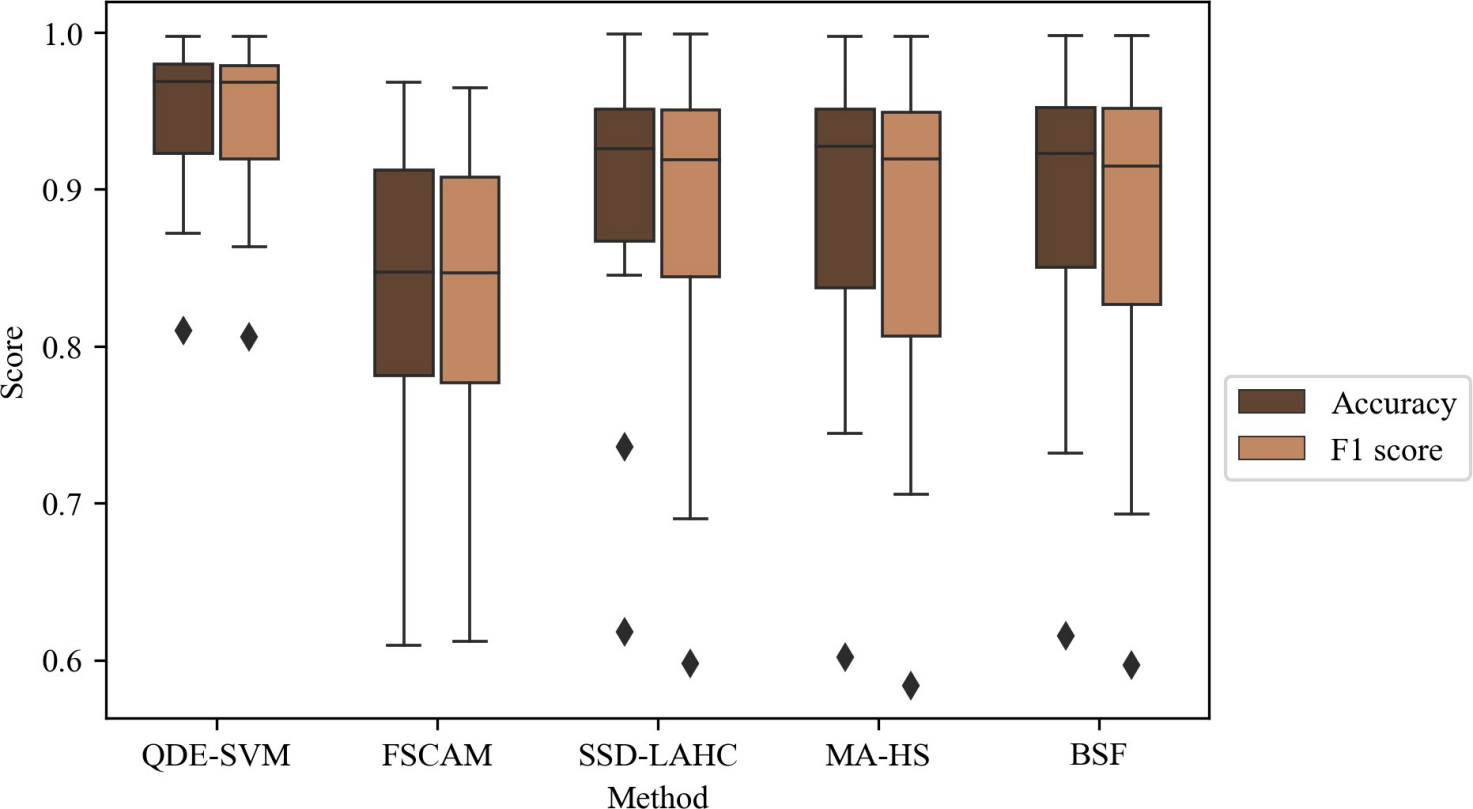
Boxplot of cell type classification accuracy and F1-score of all datasets using gene features selected by different wrapper methods.

Table 8 shows the comparison of accuracy and F1-score between QDE-SVM and the recent methods. For the classification performance of the selected features on cell type identification, QDE-SVM has higher average scores as compared to the other methods (the average of 0.9456 and 0.9429 for accuracy and F1-score respectively). This phenomenon is also observed in a boxplot in Fig 2. The gene features selected by FSCAM achieve the lowest cell type classification performance as compared to the other wrapper methods (the average of 0.8292 and 0.8258 for accuracy and F1-score respectively). On the other hand, SSD-LAHC, MA-HS, and BSF perform with a moderate classification performance within a range of average accuracy between 0.8793 and 0.8872, and a range of average F1-score between 0.8679 and 0.8752.

**Table 8:** Accuracy and F1-score from different wrapper methods.

**(A) Accuracy**					
**Dataset**	**QDE-SVM**	**FSCAM [37]**	**SSD-LAHC [65]**	**MA-HS [67]**	**BSF [66]**
Embryo_Human_Yan_2013	0.9859	0.9680	0.9113	0.9037	0.9037
Embryo_Mouse_Biase_2014	0.9176	0.9288	0.8742	0.8561	0.8576
Embryo_Mouse_Goolam_2016	0.9668	0.9350	0.8950	0.9110	0.8633
Embryo_Mouse_Deng_2014	0.9249	0.8059	0.8455	0.7795	0.8290
Pancreas_Human_Lawlor_2017	0.9688	0.6919	0.9401	0.9498	0.9498
Pancreas_Human_Xin_2016	0.9974	0.8238	0.9988	0.9975	0.9981
Pancreas_Human_Segerstolpe_2016	0.9870	0.9069	0.9713	0.9638	0.9760
Pancreas_Human_Baron_2016	0.9776	0.8777	0.9580	0.9541	0.9582
Pancreas_Mouse_Baron_2016	0.9685	0.8663	0.9491	0.9438	0.9422
Brain_Human_Darmanis_2015	0.8721	0.6095	0.7361	0.7446	0.7317
Brain_Mouse_Zeisel_2015	0.9703	0.8286	0.9488	0.9458	0.9501
Brain_Mouse_Tasic_2016	0.8101	0.7076	0.6178	0.6016	0.6155
***Average accuracy***	***0*.*9456***	***0*.*8292***	***0*.*8872***	***0*.*8793***	***0*.*8813***
**(B) F1-score**					
**Dataset**	**QDE-SVM**	**FSCAM [37]**	**SSD-LAHC [65]**	**MA-HS [67]**	**BSF [66]**
Embryo_Human_Yan_2013	0.9843	0.9648	0.9021	0.8918	0.8925
Embryo_Mouse_Biase_2014	0.9062	0.9152	0.8419	0.8175	0.8258
Embryo_Mouse_Goolam_2016	0.9638	0.9286	0.8714	0.8982	0.8379
Embryo_Mouse_Deng_2014	0.9239	0.8015	0.8448	0.7725	0.8267
Pancreas_Human_Lawlor_2017	0.9681	0.6898	0.9360	0.9484	0.9456
Pancreas_Human_Xin_2016	0.9974	0.8207	0.9988	0.9975	0.9981
Pancreas_Human_Segerstolpe_2016	0.9863	0.9052	0.9698	0.9621	0.9746
Pancreas_Human_Baron_2016	0.9771	0.8752	0.9566	0.9520	0.9570
Pancreas_Mouse_Baron_2016	0.9679	0.8645	0.9454	0.9400	0.9373
Brain_Human_Darmanis_2015	0.8634	0.6120	0.6900	0.7055	0.6930
Brain_Mouse_Zeisel_2015	0.9703	0.8286	0.9484	0.9456	0.9498
Brain_Mouse_Tasic_2016	0.8058	0.7032	0.5976	0.5837	0.5970
***Average F1-score***	***0*.*9429***	***0*.*8258***	***0*.*8752***	***0*.*8679***	***0*.*8696***

In terms of the number of selected gene features, QDE-SVM, SSD-LAHC, and BSF have obtained nearly thirteen thousand gene features on average (Table 9). They might not be favorable for use in applications such as probes design in spatial transcriptomics wherein a much smaller set of gene features is required [35]. On the other hand, the average number of features obtained by MA-HS (an average unit of 4478.12 genes) is between those of FSCAM, QDE-SVM, and SSD-LAHC. The number of features selected by FSCAM is the smallest among all, with only an average unit of 531.40 genes. In short, FSCAM is a better candidate than the rest in obtaining a small number of genes for classifying cell types.

**Table 9:** Number of selected features from different wrapper methods.

Dataset	QDE-SVM	FSCAM [37]	SSD-LAHC [65]	MA-HS [67]	BSF [66]
Embryo_Human_Yan_2013	7148.93	333.00	7095.80	2254.40	5113.20
Embryo_Mouse_Biase_2014	12733.49	749.40	12655.40	2973.40	10253.60
Embryo_Mouse_Goolam_2016	20578.70	464.00	20464.60	5474.20	9704.60
Embryo_Mouse_Deng_2014	11406.62	699.40	11453.40	3856.00	6858.00
Pancreas_Human_Lawlor_2017	13251.16	238.40	13122.40	5021.40	16816.60
Pancreas_Human_Xin_2016	19819.90	234.00	19615.20	6004.00	16541.80
Pancreas_Human_Segerstolpe_2016	13055.55	262.60	13035.60	5210.40	15624.00
Pancreas_Human_Baron_2016	9970.22	467.60	10023.40	3729.60	12016.20
Pancreas_Mouse_Baron_2016	7385.53	372.20	7456.80	3538.80	9984.20
Brain_Human_Darmanis_2015	11018.63	327.80	11020.00	3920.00	15303.00
Brain_Mouse_Zeisel_2015	9916.18	235.00	9956.60	3147.80	11943.20
Brain_Mouse_Tasic_2016	12059.45	1993.40	12018.40	8607.40	20282.60
***Average number of selected features***	***12362*.*03***	***531*.*40***	***12326*.*47***	***4478*.*12***	***12536*.*75***

On the other hand, as shown in Table 10, the time taken by FSCAM is much shorter for small datasets. However, when compared with QDE-SVM, such advantage of FSCAM is not shown when processing the datasets with large sample sizes such as Pancreas_Xin_2016, Pancreas_Segerstolpe_2016, Pancreas_Human_Baron_2016, Pancreas_Mouse_Baron_2016, and Brain_Mouse_Zeisel_2015. The time needed for SSD-LAHC, MA-HS, and BSF is much longer (on average from 169.65 to 295.15 hours) when compared to QDE-SVM (an average time of 29.85 hours) and FSCAM (an average time of 43.18 hours). Overall, QDE-SVM has the shortest time of execution on average.

**Table 10:** Time taken (in hours) for feature selection using different wrapper methods.

Dataset	QDE-SVM	FSCAM [37]	SSD-LAHC [65]	MA-HS [67]	BSF [66]
Embryo_Human_Yan_2013	1.02	0.59	24.34	50.43	82.70
Embryo_Mouse_Biase_2014	3.20	0.58	78.06	96.72	134.12
Embryo_Mouse_Goolam_2016	4.50	0.99	45.78	128.12	172.09
Embryo_Mouse_Deng_2014	7.79	2.21	29.06	80.19	117.78
Pancreas_Human_Lawlor_2017	9.34	6.56	58.41	143.40	213.93
Pancreas_Human_Xin_2016	25.17	67.16	343.55	301.75	538.02
Pancreas_Human_Segerstolpe_2016	46.83	69.49	197.74	221.17	418.92
Pancreas_Human_Baron_2016	103.33	230.43	726.91	769.41	913.27
Pancreas_Mouse_Baron_2016	15.17	46.47	64.62	127.49	170.97
Brain_Human_Darmanis_2015	16.10	2.66	72.15	89.51	120.32
Brain_Mouse_Zeisel_2015	86.31	72.38	129.52	162.67	363.37
Brain_Mouse_Tasic_2016	39.45	18.64	265.62	233.25	296.27
***Average time (hours)***	***29*.*85***	***43*.*18***	***169*.*65***	***200*.*34***	***295*.*15***

Statistical tests on results from different wrapper methods were conducted using Friedman test and followed by the post hoc tests using Holm’s procedure to examine their significance. As usual, an *α* value of 0.05 was used as the significant level to test for the null hypothesis that all methods give statistically similar results. By referring to the results in Table 11, the null hypotheses are rejected with lower *p*-values for the tests on accuracy, F1-score, number of features, and time taken.

**Table 11:** Friedman tests for 5 different wrapper-based methods (QDE-SVM, FSCAM, SSD-LAHC, MA-HS, and BSF) in terms of accuracy, F1-score, number of features, and time taken.

Comparison	Test statistic	*p*-value	Hypothesis
Five wrappers (Accuracy)	19.97	5.07E-04	Rejected
Five wrappers (F1-score)	19.53	6.17E-04	Rejected
Five wrappers (Number of features)	38.67	8.16E-08	Rejected
Five wrappers (Time taken)	44.53	4.97E-09	Rejected

In the post hoc tests, QDE-SVM shows significant differences in cell type classification accuracy and F1-score among all other methods (Table 12). This implies that it can achieve better classification performance than the other methods. On the other hand, the number of gene features obtained by QDE-SVM is statistically similar to those of SSD-LAHC and BSF, and is significantly greater than those of FSCAM and MA-HS. The post hoc test results on execution times indicate that only QDE-SVM and FSCAM have a similar time of execution when processing all datasets. The other three wrapper methods utilize much longer execution times.

**Table 12:** Post hoc tests among different wrapper-based methods (QDE-SVM as the control algorithm) using Holm procedure.

**(A) Accuracy of cell type classification using selected features**					
***i***	**QDE-SVM *vs*.**	***z***	***p*-value**	**Adjusted α**	**Hypothesis**
1	FSCAM	-4.7434	0.0000	0.0125	Rejected
4	SSD-LAHC	-2.8460	0.0044	0.0500	Rejected
2	MA-HS	-4.6644	0.0000	0.0167	Rejected
3	BSF	-3.5576	0.0004	0.0250	Rejected
**(B) F1-score of cell type classification using selected features**					
***i***	**QDE-SVM *vs*.**	***z***	***p*-value**	**Adjusted α**	**Hypothesis**
1	FSCAM	-4.7434	0.0000	0.0125	Rejected
4	SSD-LAHC	-3.0042	0.0027	0.0500	Rejected
2	MA-HS	-4.5853	0.0000	0.0167	Rejected
3	BSF	-3.4785	0.0005	0.0250	Rejected
**(C) Number of selected features**					
***i***	**QDE-SVM *vs*.**	***z***	***p*-value**	**Adjusted α**	**Hypothesis**
1	FSCAM	-5.6921	0.0000	0.0125	Rejected
4	SSD-LAHC	-0.3162	0.7518	0.0500	Accepted
2	MA-HS	-3.7947	0.0001	0.0167	Rejected
3	BSF	0.3162	0.7518	0.0250	Accepted
**(D) Time taken for feature selection**					
***i***	**QDE-SVM *vs*.**	***z***	***p*-value**	**Adjusted α**	**Hypothesis**
4	FSCAM	-0.6325	0.5271	0.0500	Accepted
3	SSD-LAHC	2.8460	0.0044	0.0250	Rejected
2	MA-HS	4.1110	0.0000	0.0167	Rejected
1	BSF	6.3246	0.0000	0.0125	Rejected

In summary, QDE-SVM is statistically more accurate than FSCAM, MA-HS, SSD-LAHC, and BSF in classifying cell types at the expense of utilizing a greater number of gene features than FSCAM and MA-HS. The number of gene features obtained by QDE-SVM is statistically the same as those of SSD-LAHC and BSF. Its execution time is as fast as FSCAM and is much faster than MA-HS, SSD-LAHC, and BSF. FSCAM applies pre-filtering of genes before running feature selection with FFOA. This explains the reason why the number of features selected by FSCAM is much lesser than all the other methods which do not filter any of the genes in advance.

## Discussion

To further validate and analyze the effectiveness of gene features selected by the proposed method, the best solution (gene feature subset) was extracted from the five-fold candidate solutions of each method. For each method, the gene subset with the highest fitness score (as defined in Eq (1)) was identified as the best solution. The gene overlapping rates of the best solution obtained by the proposed method with the other four methods were examined to find further insights. The percentage of overlapping was calculated using the Jaccard score [70]. Table 13 shows the number and percentage of overlapping genes between the gene subsets from QDE-SVM and the other wrapper-based methods. Note that little genes from QDE-SVM are overlapped with other wrapper methods (in a range from 0.32% to 45.11%). This shows that the genes selected using the five methods are different, and this phenomenon can also be observed in other gene selection studies [71, 72]. The number of overlapping genes between QDE-SVM and FSCAM is relatively lesser as compared to QDE-SVM and the other three methods. The reason is that, FSCAM selects the smallest number of genes, and not many of these genes are also selected by QDE-SVM. On the other hand, the genes that are not selected by other methods but by QDE-SVM might be the key genes contributing to the higher classification accuracy in QDE-SVM.

**Table 13:** Number and percentage of overlapping genes between QDE-SVM and other wrapper-based methods.

**(A) Number of genes overlap with QDE-SVM**				
**Dataset**	**FSCAM**	**SSD-LAHC**	**MA-HS**	**BSF**
Embryo_Human_Yan_2013	107	3474	919	508
Embryo_Mouse_Biase_2014	380	6228	1364	835
Embryo_Mouse_Goolam_2016	141	10025	2439	1340
Embryo_Mouse_Deng_2014	145	5583	1764	1456
Pancreas_Human_Lawlor_2017	90	6465	1667	8268
Pancreas_Human_Xin_2016	62	9605	2199	6206
Pancreas_Human_Segerstolpe_2016	151	6381	1965	7491
Pancreas_Human_Baron_2016	239	4888	1213	5740
Pancreas_Mouse_Baron_2016	162	3620	1307	4762
Brain_Human_Darmanis_2015	89	5404	1390	7588
Brain_Mouse_Zeisel_2015	68	4857	1297	3124
Brain_Mouse_Tasic_2016	823	5930	2043	9806
**(B) Percentage of genes overlap with QDE-SVM (%)**				
**Dataset**	**FSCAM**	**SSD-LAHC**	**MA-HS**	**BSF**
Embryo_Human_Yan_2013	1.50	32.58	11.48	6.70
Embryo_Mouse_Biase_2014	2.94	32.78	9.81	6.22
Embryo_Mouse_Goolam_2016	0.69	32.62	10.69	6.15
Embryo_Mouse_Deng_2014	1.26	32.84	13.36	11.34
Pancreas_Human_Lawlor_2017	0.68	32.51	11.22	38.07
Pancreas_Human_Xin_2016	0.32	32.46	10.04	23.89
Pancreas_Human_Segerstolpe_2016	1.15	32.70	13.19	36.26
Pancreas_Human_Baron_2016	2.37	32.92	10.96	36.22
Pancreas_Mouse_Baron_2016	2.16	32.64	15.15	39.06
Brain_Human_Darmanis_2015	0.81	32.93	11.32	40.61
Brain_Mouse_Zeisel_2015	0.69	32.47	11.63	23.99
Brain_Mouse_Tasic_2016	6.37	32.67	14.47	45.11

As the genes selected by the five methods are different, the biological significance of the selected gene subsets is further discussed after conducting a gene enrichment analysis. Gene Ontology (GO) enrichment analysis was performed at http://geneontology.org/ to validate the biological significance of the selected gene subset. The test and correction methods used for the enrichment analyses were Fisher’s exact test and false discovery rate (FDR). For each gene subset, the top 15 enriched GO terms were determined from terms of third-level and above in the ontology, as well as terms with high gene ratios and low FDR. For each category of the datasets (developmental, metabolic diseases, and connective tissues), a dataset with a moderate number of genes was chosen as the representative dataset for analysis (Embryo_Mouse_Biase_2014 to represent embryo development datasets, Pancreas_Human_Segerstolpe_2016 to represent pancreas tissue and metabolic disease datasets, and Brain_Human_Darmanis_2015 to represent connective datasets).

**Fig 3:**
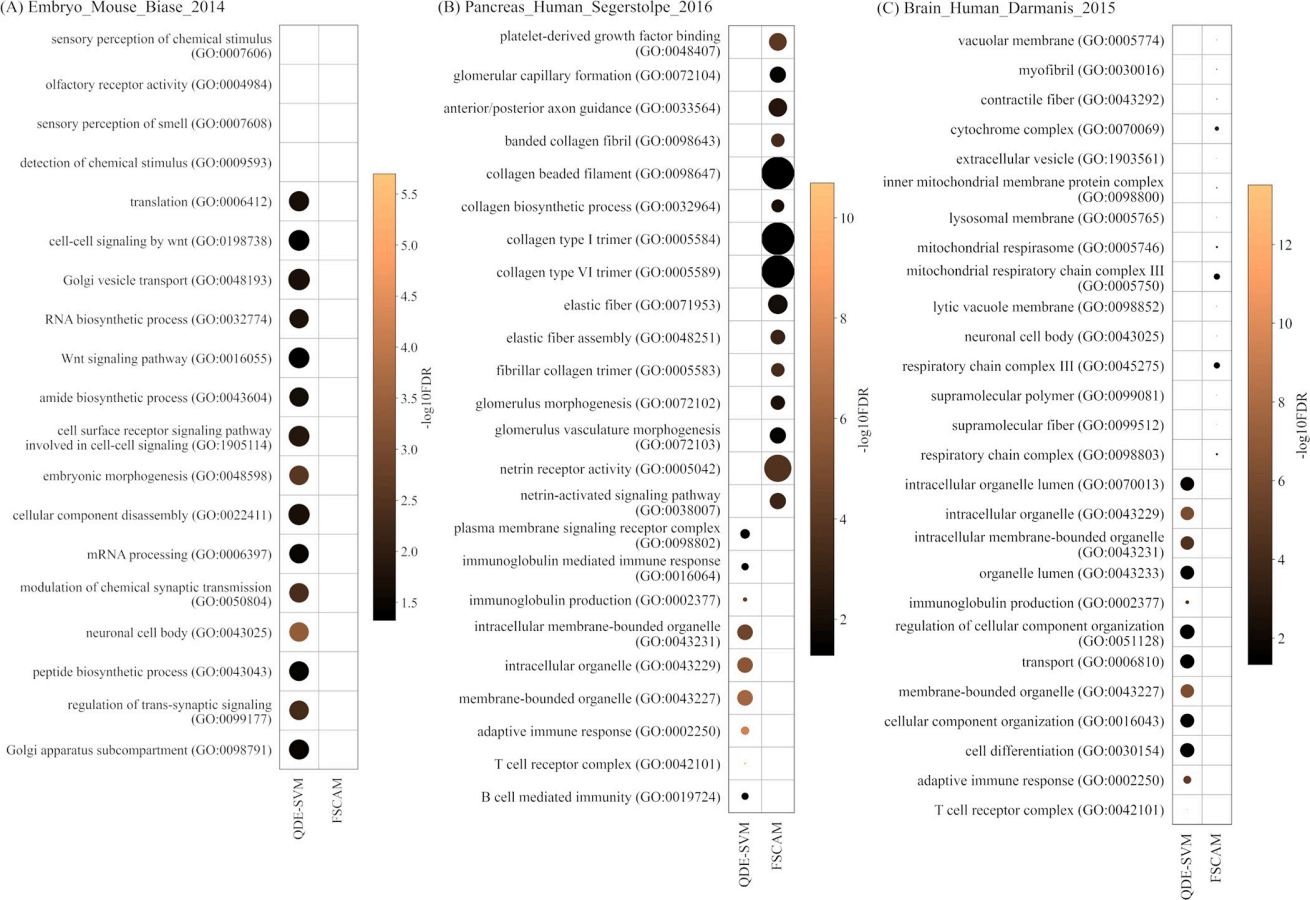
GO enrichment results of gene subsets from QDE-SVM and FSCAM for the datasets (A) Embryo_Mouse_Biase_2014 (B) Pancreas_Human_Segerstolpe_2016, and (C) Brain_Mouse_Darmanis_2015.

Fig 3A–3C show GO enrichment results of the gene subsets from QDE-SVM for the datasets Embryo_Mouse_Biase_2014, Pancreas_Human_Segerstolpe_2016, and Brain_Mouse_Darmanis_2015 respectively. Gene subsets from FSCAM were also included in the enrichment analysis for reference, as it is also a wrapper-based method introduced for scRNA-seq gene selection. The horizontal axis shows the feature selection methods, while the vertical axis shows the enriched GO terms. The ratio of genes in the gene subset that matched with the genes involved in a GO term is represented by the data point size. The larger the point, the more genes matched the terms. The color intensity of the data points represents -log_10_FDR, where the lighter color shows lower FDR.

For the embryo development dataset (Embryo_Mouse_Biase_2014), the gene subset selected by QDE-SVM is enriched in developmental-related terms such as Golgi vesicle transport, which are essential for embryo development [73]. Signaling pathways are also found to be important in embryogenesis for the secretion of essential proteins such as growth factors [74, 75]. Other terms such as mRNA processing, translation, embryonic morphogenesis, and cellular component disassembly are related to the experiment setup of Embryo_Mouse_Biase_2014 as well (Fig 3A). For the gene subset from FSCAM, very few genes are mapped to the four enriched GO terms most probably due to a small number of genes being selected. It is unlikely that one could obtain any valuable biological insights from this result. Related GO terms can also be seen in the Pancreas_Human_Segerstolpe_2016 gene subsets (Fig 3B). For the gene subset from QDE-SVM, the enriched terms include intracellular organelle, such as mitochondria, which is important for insulin regulation. Intracellular organelle stress is found to be one of the potential research directions for T2D treatments [76]. Other than that, the analysis shows immunology-related terms are enriched in the gene subset. This can be related to the presence of immune cells in the pancreas as reported by Wu et al. [77]. For the gene subset from FSCAM, the genes are highly enriched in collagen-related terms, mostly with all genes mapped. This might be due to the presence of a large amount of collagen in the pancreas extracellular matrix [77]. In addition, there are also netrin-related terms found in the gene subset from FSCAM, which could be related to pancreatic development [78]. Other terms such as glomerular-related terms may not be useful for the pancreatic dataset to our knowledge. For Brain_Mouse_Darmanis_2015 dataset, the enriched terms identified for the gene subset from QDE-SVM are mostly related to organelles (Fig 3C), which might be related to the essential cell functions. There is also a small portion of immunology-related terms. T cells are found to be important for CNS neuroprotection [79], thus, this might explain why there are T cells receptor complex and adaptive immune response terms in the cortex cells. The rest of the terms include cell differentiation, which is important for generating various cells in the brain. For the gene subset from FSCAM, the gene ratio is relatively low due to fewer genes in the gene subset. However, they are also somehow related to the dataset. For example, mitochondria and respiratory chain complex-related terms might be involved in the brain aging process and neurodegenerative diseases [80, 81]. Other terms are also somehow related to the brain tissue such as fibers and lysozymes [82].

GO enrichment analysis shows that the genes selected by QDE-SVM are biologically relevant. The top 15 terms are not similar to the one in FSCAM, which is expected as the overlapping genes are few. Both methods could be useful in discovering biologically relevant genes. Overall, this study provides a brief functional analysis of the gene features selected. More practical efforts are needed to validate the usefulness of the GO terms in downstream applications such as biomarker design.

QDE-SVM is potentially useful in various applications. While serving for reducing the scRNA-seq data dimension, the selected gene features can also be applied for future cell-type classification tasks in similar experimental settings [23]. As not all of the genes are useful for classification tasks, selecting genes that contribute to accurate classification helps to assign cell types correctly for the newly-sequenced single cells. Besides, the gene selected could also facilitate the downstream identification of marker genes [7, 35]. The marker genes could be used to distinguish cell types, cell stages, certain diseases, or conditions. Reducing the number of potential marker genes using QDE-SVM eases laboratory experiments or tests.

The classification component in QDE-SVM gives a slightly different observation of results from the clustering-based method. It is noticed that classification-based methods (i.e., QDE-SVM, SSD-LAHC, MA-HS, and BSF) generally select more accurate gene subsets than the clustering-based method (i.e., FSCAM). This is reasonable as they are supervised algorithms provided with labeled cells. However, the number of genes selected by QDE-SVM is only around half of the original number of genes, which might still be further reduced for feasible downstream applications. Also, when comparing different feature selection categories, an obvious limitation of the wrapper-based method is the time needed to conduct feature selection. The well-known non-wrapper-based methods such as Seurat [9] or scmap [23] fall under the filter-based feature selection category. Filter-based feature selection methods have an advantage of fast execution. This also explains why there are fewer works published on wrapper-based methods as the computational time required will increase with the number of iterations. Nevertheless, wrapper-based methods impose learning algorithms to assess the quality of features during iterative search process, and this contributes to finding better (more accurate) feature subsets. Filter-based methods that select gene features prior to assessment with learning algorithm might not be accurate enough for downstream applications such as cell type classification [22]. Thus, the proposed wrapper-based method could still be useful with several improvements.

In the future, the first effort should be taken to reduce the number of genes while still preserving the superior classification performance of QDE-SVM. It can be done using threshold-based filtering steps as in FSCAM or using other filtering methods such as information theory, distances, correlation, etc. This would require additional studies and experiments to determine the suitable filters. Another possible future work is to improve QDE using different schemes of mutation and crossover [83] or different selection strategies [84, 85], so that it could be more explorative when searching subsets in the large feature space. Additionally, the performance of the selected gene features could also be tested across datasets with similar experimental settings, such as from the same tissue, disease, or sequencing platform and protocol. This is a key step to move forward to the application stage, i.e., cell-type classification.

## Conclusion

In conclusion, a classification-based wrapper method for scRNA-seq gene selection has been presented. A linear SVM wrapped with QDE was suggested in this work based on the feature selection results on twelve well-known scRNA-seq transcriptomic data. QDE-SVM has been tested and validated to select biologically relevant gene subsets with superior cell type classification performance when compared to QDE wrapping with other classifiers and the recent wrapper methods. However, QDE-SVM has a limitation when compared to a recent wrapper-based scRNA-seq gene selection method, FSCAM. The number of features being selected by QDE-SVM could still be reduced to obtain a set of informative marker genes for effective downstream analyses. Nevertheless, given the higher accuracy achieved by QDE-SVM in a similar time required for both of the wrapper methods, QDE-SVM is suggested as a promising gene selection method for further exploration.
